# Risk Factors Associated With Febrile Seizures in Young Children: Clinical, Biochemical, and Genetic Perspectives

**DOI:** 10.7759/cureus.109571

**Published:** 2026-05-24

**Authors:** Harikeshwar Mishra, Shakal N Singh, Manisha Verma, Areesha Alam, Chandrakanta Kumar, Himanshu Gupta, Mohammad K Ahmad

**Affiliations:** 1 Department of Pediatrics, Autonomous State Medical College, Kushinagar, IND; 2 Department of Pediatrics, King George's Medical University, Lucknow, IND; 3 Department of Biochemistry, King George's Medical University, Lucknow, IND

**Keywords:** complex seizure, early childhood, febrile seizures, kcc2 gene polymorphism, risk factors

## Abstract

Background and objective

Febrile seizures are the most common seizure disorder in children under five years of age. Although typically benign, their recurrence and atypical presentations warrant a deeper understanding of underlying risk factors. This study aims to explore the clinical, biochemical, and genetic factors associated with febrile seizures in young children.

Methodology

A hospital-based case-control investigation was conducted at a tertiary care teaching hospital in Uttar Pradesh. A total of 106 children aged six months to five years were enrolled, including 53 cases with febrile seizures and 53 age- and sex-matched febrile controls without seizures. KCC2 gene polymorphism analysis (rs2297201) was performed in children with atypical or recurrent febrile seizures and in matched controls. Comparative analyses were performed using the chi-squared test and Student's t-test (two-tailed), with a p-value of < 0.05 regarded as statistically significant.

Results

A total of 40 patients out of 53 (75.47%) experienced simple seizures, while 13 (24.53%) had complex seizures. Recurrence was reported in 19 (35.85%) cases out of 53. Electrolyte and biochemical analysis revealed significantly lower sodium, ionized calcium, magnesium, iron, vitamin B12, and vitamin D levels in the case group. Hemoglobin and mean corpuscular volume (MCV) were also significantly lower in the case group (p < 0.0001). Genotype analysis of the KCC2 rs2297201 polymorphism revealed a higher frequency of the CC genotype in the case group (p = 0.0318).

Conclusion

This study identifies significant clinical, biochemical, and genetic differences between children with febrile seizures and febrile controls. These findings may contribute to understanding the underlying mechanisms of febrile seizures and help identify potential biomarkers for early detection.

## Introduction

Febrile seizures are the most common type of convulsive event in children under five years, with a prevalence of approximately 2-5% among otherwise neurologically healthy infants and young children [1]. They are defined as seizures associated with a body temperature above 38°C (100.4°F), occurring between 6 and 60 months of age, without evidence of central nervous system infection, metabolic disturbances, or a prior history of afebrile seizures [2].

Febrile seizures are broadly categorized into simple (typical) and complex (atypical) types. Simple febrile seizures are generalized tonic-clonic in nature, last for 15 minutes or less, and do not recur within 24 hours in neurologically intact children [3]. In contrast, complex febrile seizures are prolonged (lasting more than 15 minutes), may recur within 24 hours, exhibit focal features, or occur in children with pre-existing neurological abnormalities or postictal deficits [3].

While febrile seizures are generally benign and self-limiting, their recurrence is a significant concern for both clinicians and parents. Approximately 30% of children experience a recurrence after the first episode, and this increases to 50% after a second episode [4]. Identified risk factors for recurrence include early age at onset (<15 months), a positive family history of febrile seizures or epilepsy, shorter interval between fever onset and seizure, lower peak body temperature, male sex, and reduced serum sodium levels at presentation [5].

The pathophysiology of febrile seizures is multifactorial, involving a combination of genetic, environmental, and biochemical factors [6]. Micronutrient deficiencies - particularly of iron, calcium, magnesium, folic acid, vitamin B12, and vitamin D - have been implicated as potential contributing factors [7]. Furthermore, emerging genetic studies suggest a possible role of gene polymorphisms, including variations in the KCC2 gene (rs2297201), which encodes a neuron-specific potassium-chloride cotransporter involved in neuronal excitability and seizure susceptibility [8].

Additionally, growing evidence suggests that inflammatory mediators and cytokine responses during febrile illnesses may lower the seizure threshold. Alterations in neuronal ion channel function and neurotransmitter imbalance, particularly involving GABAergic and glutamatergic pathways, further contribute to increased neuronal excitability. Advances in molecular genetics and biomarker research are providing new insights into susceptibility patterns and may facilitate early risk prediction and personalized management strategies in affected children.

The majority of febrile seizures occur during viral illnesses, especially upper respiratory tract infections, otitis media, and gastroenteritis, with peak incidence noted in winter and during the late afternoon or evening. Seasonal and circadian variations in incidence further highlight the complex interplay of environmental triggers [9].

Despite the benign nature of febrile seizures, they are a common reason for emergency pediatric admissions worldwide. Parental anxiety often stems from fears of recurrence or the development of epilepsy later in life [10,11]. Understanding the clinical, biochemical, and genetic risk factors associated with febrile seizures is critical for early identification of high-risk children, appropriate management, and parental counseling.

The objective of this study is to evaluate the clinical risk factors associated with febrile seizures in children under five years of age, with a focus on identifying key predictors of recurrence and atypical presentation.

## Materials and methods

A hospital-based case-control study was conducted in the Department of Pediatrics at King George's Medical University (KGMU), Lucknow, from October 2022 to September 2023, following approval from the Institutional Ethics Committee. Subjects were recruited from both the pediatrics outpatient department (OPD) and admitted patients in the pediatric emergency department. Newly diagnosed cases of febrile seizures were enrolled after obtaining written informed consent from parents or legal guardians.

The study included a total of 106 children aged six months to five years, comprising 53 cases with febrile seizures and 53 age and sex matched febrile controls without seizures. For the case group, participants must be diagnosed with febrile seizures, while for the control group, children must have a fever without experiencing seizures. Children were excluded if they had known neurodevelopmental disorders, developmental delay, epilepsy syndromes, or central nervous system infections, such as meningitis or encephalitis.

The sample size was calculated based on data from prior studies by Aydin et al. (2021) [12] and AL-Zwaini (2006) [13]. Based on an assumed exposure proportion of 0.20 in the control group and 0.33 in the case group, with an anticipated odds ratio of 2, 80% statistical power, and a two-sided alpha of 0.10, the calculated sample size was 53 participants per group.

Demographic and clinical information were documented using a pre-designed proforma. Subjects were followed during the study period for seizure recurrence or development of atypical features. All participants underwent the blood investigations including complete blood count (CBC), serum electrolytes that included sodium (Na⁺), calcium (Ca²⁺), magnesium (Mg²⁺), blood glucose levels (BGL), arterial/capillary blood gas (ABG/CBG) (pH, PCO₂, HCO₃⁻), serum 25(OH) vitamin D, serum vitamin B12, serum folic acid, and serum iron. Molecular studies were conducted on children with atypical or recurrent febrile seizures and an equal number of matched controls. The gene studied was KCC2 (rs2297201). To study the polymorphism of the KCC2 gene, 2 ml of peripheral blood sample was collected in an ethylenediaminetetraacetic acid (EDTA) vial. DNA extraction was performed by the organic extraction method. DNA was stored at -80°C for future use.

Polymerase chain reaction (PCR) was performed using gene-specific primers, and gene polymorphisms were identified using the restriction fragment length polymorphism (RFLP) method.

Data were entered into Microsoft Excel (Microsoft, Redmond, Washington) and analyzed using SPSS version 26 (IBM Inc., Armonk, New York). Continuous variables are presented as mean ± standard deviation (SD) and were compared using the Student's t-test, while categorical variables are expressed as frequencies and percentages and were analyzed using the chi-squared test. Statistical significance was set at p < 0.05.

## Results

A total of 106 children were enrolled, with 53 cases (children with febrile seizures) and 53 age- and sex-matched controls (febrile children without seizures). Table 1 shows demographic characteristics of cases and controls.

**Table 1:** Demographical profile of the patients among the case and control group

Parameter	Category	Case (N=53)	Control (N=53)	Test value	p-value
Age (months) (mean ± SD)		20.41 ± 5.42	21.34 ± 5.74	t = 0.8576	0.3931
Gender	Female	30 (56.60%)	25 (47.17%)	χ² = 0.7984	0.4587
	Male	23 (43.40%)	28 (52.83%)	—	—
Residence	Rural	40 (75.47%)	37 (69.81%)	χ² = 0.4272	0.5134
	Urban	13 (24.53%)	16 (30.19%)	—	—
Diet	Vegetarian	18 (33.96%)	14 (26.42%)	χ² = 0.7162	0.3974
	Non-vegetarian	35 (66.04%)	39 (73.58%)	—	—
Weight (kg) (Mean ± SD)		11.93 ± 2.53	11.65 ± 2.42	t = 0.5822	0.5617
Height (cm) (Mean ± SD)		82.42 ± 10.42	82.57 ± 10.74	t = 0.07298	0.942

The mean age of participants in the case group was 20.41 ± 5.42 months, compared to 21.34 ± 5.74 months in the control group, with no statistically significant difference between the groups (p = 0.3931). Gender distribution was comparable between groups (p = 0.4587), with a slight female predominance among cases. Residence status showed a higher proportion of rural dwellers in both groups, though the difference was not statistically significant (p = 0.5134). Dietary pattern also did not differ significantly between groups (p = 0.3974), with a predominance of a non-vegetarian diet in both. Anthropometric parameters, including mean weight and height, were comparable between the groups (p = 0.5617 and p = 0.9420, respectively), indicating no significant differences in nutritional status. These findings demonstrate that the groups were well matched in terms of demographic and baseline clinical characteristics, thereby minimizing potential confounding from these variables.

Tables 2 and Table 3 present the comparison of patient history and clinical parameters between the case and control groups, respectively. No statistically significant differences were observed across various history parameters between the two groups.

**Table 2:** Patient history among the case and control groups

History parameter	Case, n (%)	Control, n (%)	p-value
Family history of febrile seizure	8 (15.09%)	2 (3.77%)	p = 0.3498
History of head trauma	2 (3.77%)	2 (3.77%)	> 0.9999
History of iron folic acid supplementation	10 (18.87%)	6 (11.32%)	p = 0.7224
History of calcium & vitamin D supplementation	12 (22.64%)	9 (16.98%)	p = 0.7579

**Table 3:** Comparison of clinical parameters among the case and control groups *p < 0.05 considered statistically significant

Clinical parameter	Case (n=53)	Control (n=53)	Mean difference	Test statistic	p-value
Heart rate (beats/min) (mean ± SD)	134.53 ± 20.60	126.45 ± 18.67	-8.08	t = 2.116	0.0367*
Respiratory rate (breaths/min) (mean ± SD)	30.51 ± 8.51	28.67 ± 7.12	1.84	t = 0.01574	0.9875
SpO₂ (%) (mean ± SD)	94.24 ± 4.54	95.47 ± 3.85	1.43	t = 1.749	0.0833
Temperature (°F) (mean ± SD)	101.54 ± 0.43	101.73 ± 0.49	0.19	t = 2.122	0.0362*
Blood glucose level (mg/dL) (mean ± SD)	91.46 ± 10.54	94.84 ± 11.24	3.58	t = 1.691	0.0937
Pallor	10 (18.87%)	8 (15.09%)	—	χ² = 0.2677	0.6051

A significant difference in heart rate was observed between the two groups. The mean temperature in the case group was slightly lower (101.54 ± 0.43) compared to the control group (101.73 ± 0.49), and this difference was statistically significant (p = 0.0362). Other parameters, including respiratory rate, SpO₂, blood glucose levels, and pallor, did not show statistically significant variations.

A total of 40 patients out of 53 (75.47%) experienced simple seizures, while 13 (24.53%) had complex seizures. Additionally, recurrence of febrile seizures was reported in 19 (35.85%) cases out of 53. Eleven cases out of 40 (27.5%) of simple febrile seizures had recurrence, and eight cases out of 13 (61.5%) of complex febrile seizures had recurrence. This distribution highlights that simple seizures were the most common type observed, followed by complex seizures, and a significant portion of the patients experienced a recurrence of febrile seizures. Recurrence rate was much more common in complex febrile seizures than in simple febrile seizures.

Table 4 compares the mean electrolyte levels and complete hemogram parameters between the case and control groups. For sodium, the case group had a slightly lower mean (136.12 ± 2.34) compared to the control group (137.21 ± 3.12), and this difference was statistically significant (p = 0.0444). A significant difference was also observed when looking at the percentage of patients with serum sodium levels less than 135 mmol/l; 35 patients (66.04%) had serum sodium levels less than 135 mmol/l in the case group compared to 13 patients (24.53%) in the control group, with a p-value < 0.0001.

**Table 4:** Comparison of mean electrolyte levels and complete hemogram among case and control patients *p < 0.05 considered statistically significant

Electrolyte/parameter	Case (n=53)	Control (n=53)	Mean difference	Test statistic	p-value
Serum sodium (mmol/l) (mean ± SD)	136.12 ± 2.34	137.21 ± 3.12	1.09	t = 2.035	p = 0.0444*
Serum sodium < 135 (n, %)	35 (66.04%)	13 (24.53%)	X = 18.43	χ² = 18.43	p < 0.0001*
Serum ionized calcium (mg/dl) (mean ± SD)	3.51 ± 2.23	4.45 ± 2.47	0.94	t = 2.056	p = 0.0422*
Serum ionized calcium < 1.35 (n, %)	40 (75.47%)	10 (18.87%)	X = 34.07	χ² = 34.07	p < 0.0001*
Serum magnesium (mg/dl) (mean ± SD)	1.81 ± 0.45	1.84 ± 0.44	0.03	t = 0.3470	p = 0.7293
Serum magnesium < 1.1 (n, %)	38 (71.70%)	8 (15.09%)	X = 34.57	χ² = 34.57	p < 0.0001*
Arterial blood gas pH (mean ± SD)	7.42 ± 0.12	7.40 ± 0.21	-0.02	t = 0.6020	p = 0.5485
Arterial blood gas pH > 7.45 (n, %)	14 (26.41%)	6 (7.54%)	X = 0.3332	χ² = 0.3332	p = 0.3332
pCO2 (mmHg) (mean ± SD)	30.77 ± 5.33	36.41 ± 2.42	5.64	t = 7.014	p < 0.0001*
pCO2 < 35 (n, %)	35 (66.04%)	20 (37.74%)	X = 8.503	χ² = 8.503	p = 0.0035*
Blood HCO3 (mEq/L) (mean ± SD)	24.02 ± 2.98	24.21 ± 1.16	0.19	t = 0.4326	p = 0.6662
HCO3 < 22 (n, %)	5 (9.43%)	4 (7.55%)	X = 0.1214	χ² = 0.1214	p = 0.7275
Complete hemogram parameter					
Hemoglobin (Hb) (gm/dl) (mean ± SD)	11.57 ± 2.49	14.67 ± 2.64	3.10	t = 6.219	p < 0.0001*
Hb < 12 (n, %)	30 (56.60%)	15 (28.30%)	X = 8.689	χ² = 8.689	p = 0.0032*
Total leukocyte count (mean ± SD)	9800.25 ± 2500.50	9000.75 ± 2200.50	-799.3	t = 1.747	p = 0.0836
Total leukocyte count > 12000 (n, %)	15 (28.30%)	7 (13.21%)	X = 3.671	χ² = 3.671	p = 0.0554
Mean corpuscular volume (mean ± SD)	78.54 ± 8.99	81.57 ± 6.57	3.13	t = 2.046	p = 0.0432*
Mean corpuscular volume < 60 (n, %)	30 (56.60%)	8 (15.09%)	X = 19.85	χ² = 19.85	p < 0.0001*
Mean corpuscular hemoglobin (mean ± SD)	29.47 ± 3.54	29.84 ± 2.47	0.47	t = 0.7927	p = 0.4298
Mean corpuscular hemoglobin < 25 (n, %)	25 (47.17%)	7 (13.21%)	X = 14.50	χ² = 14.50	p = 0.0001*

Regarding ionized calcium, the case group had a lower mean (3.51 ± 2.23) compared to the control group (4.45 ± 2.47), with a statistically significant p-value of 0.0422. Furthermore, a significantly higher proportion of the case group, that is 40 (75.47%), had ionized calcium levels less than 1.35 mg/dl compared to the control group, that is 10 (18.87%), with a p-value of < 0.0001. For serum magnesium level, no significant difference was found (p = 0.7293), although 38 patients in the case group (71.70%) had magnesium levels less than 1.1 mg/dl compared to eight patients in the control group (15.09%), which was statistically significant (p < 0.0001).

For pCO2, the case group had a significantly lower mean (30.77 ± 5.33) compared to the control group (36.41 ± 2.42), with a p-value of < 0.0001. Additionally, 35 (66.04%) patients in the case group had pCO2 levels less than 35 mmHg, compared to 20 (37.74%) in the control group (p = 0.0035). Arterial blood gas pH and HCO3 levels showed no significant differences between the case and control groups.

For hemoglobin (Hb), the case group had a significantly lower mean (11.57 ± 2.49) compared to the control group (14.67 ± 2.64), with a mean difference of 3.10 and a p-value < 0.0001. A higher percentage of the case group (n = 30, 56.60%) had Hb levels less than 12 gm/dl compared to the control group (n = 15, 28.30%), with a statistically significant difference (p = 0.0032).

The mean corpuscular volume (MCV) was lower in the case group (78.54 ± 8.99) compared to the control group (81.57 ± 6.57), with the difference being statistically significant (p = 0.0432). Additionally, a significantly higher proportion of cases (n = 30, 56.60%) had MCV levels < 60 fL compared to controls (n = 8, 15.09%) (p < 0.0001). Although total leukocyte count (TLC) showed some variation between groups, the difference was not statistically significant.

Mean corpuscular hemoglobin (MCH) did not differ significantly between the two groups (p = 0.4298). However, a significantly higher proportion of cases (n = 25, 47.17%) had MCH levels < 25 pg/cell compared to controls (n = 7, 13.21%) (p = 0.0001).

Table 5 compares the mean biochemical parameters between the case and control groups. For iron levels, the case group had a lower mean (74.61 ± 18.34) compared to the control group (81.57 ± 15.67), with a statistically significant difference (p = 0.0381). A significantly higher proportion of patients in the case group (n = 35, 66.04%) had iron levels less than 50 mcg/dl compared to the control group (n = 10, 18.87%), with a p-value of < 0.0001.

**Table 5:** Comparison of biochemical parameters between case and control patients *p < 0.05 considered statistically significant

Biochemical parameter	Case (n=53)	Control (n=53)	Mean difference	Test statistic	p-value
Iron (mcg/dl) (mean ± SD)	74.61 ± 18.34	81.57 ± 15.67	6.96	t = 2.100	p = 0.0381*
Iron < 50 (n, %)	35 (66.04%)	10 (18.87%)	X = 24.13	χ² = 24.13	p < 0.0001*
Serum folic acid (ng/ml) (mean ± SD)	209.15 ± 33.51	211.41 ± 27.41	2.26	t = 0.3800	p = 0.7047
Serum folic acid < 150 (n, %)	15 (28.30%)	12 (22.64%)	X = 0.4473	χ² = 0.4473	p = 0.5036
Vitamin B12 (pg/ml) (mean ± SD)	310.52 ± 25.37	321.25 ± 22.49	10.73	t = 2.304	p = 0.0232*
Vitamin B12 < 300 (n, %)	38 (71.70%)	8 (15.09%)	X = 34.57	χ² = 34.57	p < 0.0001*
Vitamin D (ng/ml) (mean ± SD)	15.18 ± 7.94	17.84 ± 5.49	2.66	t = 2.006	p = 0.0474*
Vitamin D < 20 (n, %)	39 (73.58%)	14 (26.42%)	X = 23.58	χ² = 23.58	p < 0.0001*

The mean vitamin B12 level was significantly lower in the case group (310.52 ± 25.37 pg/ml) compared to the control group (321.25 ± 22.49 pg/ml) (p = 0.0232). Furthermore, a significantly higher proportion of cases (n = 38, 71.70%) had vitamin B12 levels < 300 pg/ml compared to controls (n = 8, 15.09%) (p < 0.0001). No significant difference was observed in serum folic acid levels between the groups.

Similarly, vitamin D levels were lower in the case group (15.18 ± 7.94 ng/ml) compared to the control group (17.84 ± 5.49 ng/ml), with a statistically significant difference (p = 0.0474). A higher proportion of cases (n = 39, 73.58%) had vitamin D levels < 20 ng/ml compared to controls (n = 14, 26.42%; p < 0.0001).

The distribution of KCC2 rs2297201 genotypes and allele frequencies differed between the case and control groups (Table 6) (p = 0.0318), with the TT (normal) genotype being more frequent in controls (n = 37, 69.81%) than in cases (n = 25, 47.17%). In contrast, the CC (homozygous variant) genotype was more prevalent in cases (n = 12, 22.64%) compared to controls (n = 4, 7.55%), while the TC (heterozygous) genotype showed a moderate distribution in both groups (30.19% vs. 20.75%).

The T allele was less frequent in cases (n = 33, 62.26%) compared to controls (n = 43, 81.13%), approaching statistical significance (p = 0.0513; OR = 0.3837), whereas the C allele was more common in cases (n = 20, 37.74%) than in controls (n = 10, 18.87%), suggesting a potential association with increased disease susceptibility (Table 6).

**Table 6:** KCC2 rs2297201 genotype and allele frequencies among the case and control groups *p < 0.05 considered statistically significant

Genotype and allele	Case, n (%)	Control, n (%)	p-value
Normal (TT)	25 (47.17%)	37 (69.81%)	p = 0.0318*
Homozygous (CC)	12 (22.64%)	4 (7.55%)	
Heterozygous (TC)	16 (30.19%)	12 (22.64%)	
Allele (KCC2 rs2297201)			
T allele	33 (62.26%)	43 (81.13%)	p=0.0513 OR=0.3837
C allele	20 (37.74%)	10 (18.87%)	

## Discussion

Febrile seizures (FS) represent a common neurologic phenomenon in children, typically occurring between six months and five years of age, and are generally considered benign [14]. However, the recurrence of these seizures and the occurrence of atypical presentations have raised interest in identifying the underlying risk factors that may predispose certain children to this condition. In the present study, demographic characteristics were comparable between children with febrile seizures (cases) and febrile children without seizures (controls), with no statistically significant differences observed. This includes factors such as age, gender, diet, weight, height, and residence. However, findings align with previous studies that have suggested a multifactorial and complex interplay of genetic and environmental factors in the development of febrile seizures [15,16]. Notably, family history of febrile seizures emerged as a potential risk factor, although it did not reach statistical significance, consistent with other literature highlighting the role of genetic predisposition in FS [15]. The association of febrile seizures with neurodevelopmental disorders such as autism spectrum disorder (ASD) and attention-deficit/hyperactivity disorder (ADHD) is also noteworthy, as a recent cohort study by Yuan et al. (2024) demonstrated an increased risk of these disorders in children with a history of FS [17].

Children with febrile seizures exhibited significantly elevated heart rates and lower respiratory rates, which could suggest autonomic dysregulation. These findings align with emerging research indicating that autonomic dysfunction may play a role in the pathogenesis of febrile seizures [18]. Minor differences in oxygen saturation, temperature, and blood glucose levels were observed but did not reach statistical significance. These slight deviations, while not clinically significant on their own, may reflect underlying physiological changes during febrile episodes and warrant further investigation, particularly with regard to their impact on seizure susceptibility.

When analyzing biochemical parameters, we found several significant differences between the two groups, particularly in electrolytes and micronutrient levels. Lower sodium, ionized calcium, magnesium, iron, vitamin B12, and vitamin D levels were observed in children with febrile seizures, which may have implications for the pathophysiology of the condition. These deficiencies could contribute to altered neuronal excitability, a well-established mechanism in seizure susceptibility. Specifically, lower sodium levels may be attributed to dehydration or hyponatremia associated with febrile illness, while decreased calcium levels have been linked to neuronal hyperexcitability, a known contributor to seizure risk [19]. The observed association between iron deficiency and febrile seizures is also of clinical importance, as iron deficiency has previously been linked to increased seizure risk in children [20, 21]. Furthermore, the significantly lower levels of hemoglobin and mean corpuscular volume (MCV) in the case group suggest a higher prevalence of anemia, particularly microcytic anemia, among children with febrile seizures. This finding underscores the importance of addressing hematologic abnormalities in this population, as anemia has been linked to increased susceptibility to seizures [22]. Given the high prevalence of anemia in the case group, comprehensive hemogram evaluations should be part of the clinical assessment for children presenting with febrile seizures.

The genetic analysis revealed an interesting association between the KCC2 rs2297201 polymorphism and febrile seizures. The higher prevalence of the homozygous (CC) genotype in the case group suggests a potential genetic predisposition to febrile seizures. The KCC2 gene, involved in chloride homeostasis in neurons, is critical in regulating neuronal excitability, and genetic variations in this gene may contribute to seizure susceptibility. These findings are consistent with previous studies implicating KCC2 gene polymorphisms in seizure disorders. The KCC2 rs2297201 variant may also predispose to more complex febrile seizures, as suggested by previous research linking this polymorphism to the severity of seizures [8].

The study's small sample size (53 cases and 53 controls) limits generalizability and the detection of subtle differences. Also, our study findings related to genetic differences associated with febrile seizure cannot be generalized since our study specifically evaluated the association of the KCC2 gene polymorphism. Single-center recruitment may introduce selection bias, affecting external validity. Variability in measurement techniques could lead to inconsistencies in results. Financial constraints prevented analysis of additional factors like serum copper, zinc, phosphorus, erythrocyte sedimentation rate (ESR), EEG, and MRI. Retrospective data collection may introduce recall bias, and the study may have overlooked important risk factors due to limited variable assessments. The risk of type I error increases with multiple comparisons. The febrile seizures' heterogeneous nature may not be fully captured in this study. Future research should involve larger, multi-center studies with standardized protocols, control for confounding variables, and use prospective data collection methods. Advanced statistical techniques should be applied to reduce bias, and exploration of less-studied factors like environmental exposures and neurodevelopmental abnormalities is recommended.

## Conclusions

In conclusion, the present study highlights significant clinical, biochemical, and genetic factors associated with FS in young children. Lower levels of hemoglobin, mean corpuscular volume, iron, vitamin B12, and vitamin D were found to be more prevalent in the case group, suggesting a potential link between nutritional status and seizure risk. Optimal nutrition in children can prevent the recurrence of febrile seizures. Additionally, the genotypic differences observed, particularly in the KCC2 gene, point to the involvement of genetic factors in FS pathogenesis. These findings highlight the need for further investigation into the molecular mechanisms underlying febrile seizures and underscore the importance of integrating both nutritional and genetic factors in the clinical evaluation and management of at-risk children. Ultimately, this study contributes to a better understanding of febrile seizure etiology, offering insights that may inform targeted preventive strategies and personalized therapeutic interventions for affected children.
